# Probiotic Bacteria with High Alpha-Gal Content Protect Zebrafish against Mycobacteriosis

**DOI:** 10.3390/ph14070635

**Published:** 2021-06-30

**Authors:** Iván Pacheco, Sandra Díaz-Sánchez, Marinela Contreras, Margarita Villar, Alejandro Cabezas-Cruz, Christian Gortázar, José de la Fuente

**Affiliations:** 1 SaBio, Instituto de Investigación en Recursos Cinegéticos IREC-CSIC-UCLM-JCCM, Ronda de Toledo 12, 13005 Ciudad Real, Spain; ivan.pacheco@uclm.es (I.P.); sandra.dsan@gmail.com (S.D.-S.); margaritam.villar@uclm.es (M.V.); christian.gortazar@uclm.es (C.G.); 2 Interdisciplinary Laboratory of Clinical Analysis, Interlab-UMU, Regional Campus of International Excellence Campus Mare Nostrum, University of Murcia, Espinardo, 30100 Murcia, Spain; marinelacr@hotmail.com; 3 Biochemistry Section, Faculty of Science and Chemical Technologies and Regional Centre for Biomedical Research (CRIB), University of Castilla-La Mancha, 13071 Ciudad Real, Spain; 4 UMR BIPAR, INRAE, ANSES, Ecole Nationale Vétérinaire d’Alfort, Université Paris-Est, 94700 Maisons-Alfort, France; alejandro.cabezas@vet-alfort.fr; 5 Department of Veterinary Pathobiology, Center for Veterinary Health Sciences, Oklahoma State University, Stillwater, OK 74078, USA

**Keywords:** probiotic, alpha-Gal, tuberculosis, fish, mycobacteriosis, immunology, vaccine, metabolism, antibody

## Abstract

Mycobacteriosis affects wild fish and aquaculture worldwide, and alternatives to antibiotics are needed for an effective and environmentally sound control of infectious diseases. Probiotics have shown beneficial effects on fish growth, nutrient metabolism, immune responses, disease prevention and control, and gut microbiota with higher water quality. However, the identification and characterization of the molecules and mechanisms associated with probiotics is a challenge that requires investigation. To address this challenge, herein we used the zebrafish model for the study of the efficacy and mechanisms of probiotic interventions against tuberculosis. First, bacteria from fish gut microbiota were identified with high content of the surface glycotope Galα1-3Galβ1-(3)4GlcNAc-R (α-Gal) that has been shown to induce protective immune responses. The results showed that probiotics of selected bacteria with high α-Gal content, namely *Aeromonas veronii* and *Pseudomonas entomophila*, were biosafe and effective for the control of *Mycobacterium marinum*. Protective mechanisms regulating immunity and metabolism activated in response to α-Gal and probiotics with high α-Gal content included modification of gut microbiota composition, B-cell maturation, anti-α-Gal antibodies-mediated control of mycobacteria, induced innate immune responses, beneficial effects on nutrient metabolism and reduced oxidative stress. These results support the potential of probiotics with high α-Gal content for the control of fish mycobacteriosis and suggested the possibility of exploring the development of combined probiotic treatments alone and in combination with α-Gal for the control of infectious diseases.

## 1. Introduction

The increasing incidence of infectious diseases associated with intensive aquaculture and water contamination is a major limitation for economics in aquaculture [1,2]. In particular, freshwater and marine fish mycobacteriosis caused by *Mycobacterium marinum* and other related *Mycobacterium* species affects wild fish and aquaculture [3,4]. Associated to it, the use of antibiotics has resulted in a growing prevalence of antibiotic-resistant pathogens, damage to the environment, reduced fish immunity due to effects on gut microbiota and risks associated with contaminated food [5,6,7]. Therefore, probiotics and postbiotics are considered an environmentally sustainable alternative to antibiotics for the prevention and control of infectious diseases in aquaculture.

Probiotics are live microorganisms that guide molecular interactions with potential beneficial effects to the host [8,9]. Probiotics have shown beneficial effects on fish growth, nutrient metabolism, immune responses, disease prevention and control, and gut microbiota with higher water quality [10,11]. Most probiotics used in aquaculture are lactic acid or *Bacillus* spp. due to their safety for mammalian species and production of hydrolytic enzymes that increase nutrient utilization [12,13,14]. However, recently, other criteria, such as species-specificity, pathogenicity, antibiotic resistance, extracellular enzyme production and antagonistic activity [15], have been applied for the identification of new probiotic bacteria, such as *Shewanella xiamenensis*, *Aeromonas veronii* [12], *Chromobacterium aquaticum* [16], *Streptomyces flavotricini* [17] and *Pediococcus acidilactici* [18]. Regarding fish pathogenic *Mycobacterium* spp., probiotics have shown reduction in mycobacterial levels [19,20].

One of the main challenges associated with probiotics is the identification and characterization of the molecules and mechanisms associated with its function [9]. Recently, research has been focused on the characterization of probiotic bacteria-derived postbiotic biomolecules, such as cell-wall peptidoglycans, because they are safer while retaining the beneficial effects on fish host [21]. The oligosaccharide Galα1-3Galβ1-(3)4GlcNAc-R (α-Gal) is a glycan linked to proteins and lipids in prokaryotic and eukaryotic organisms and with potential for the control of infectious diseases [22,23]. The potential of the surface glycotopes, such as α-Gal, to induce protective immune responses makes them an effective target for the development of vaccines and probiotic/postbiotic interventions [22,23,24,25,26].

The zebrafish (*Danio rerio* Hamilton 1822) has been previously validated as a fish model for the study of tuberculosis, vaccines against mycobacteriosis, fish immunity, gut microbiota and probiotics efficacy on boosting nutrient metabolism and innate immunity against pathogen infection [16,18,24,27,28,29,30,31,32,33,34,35,36]. To address the potential of α-Gal-rich probiotics for the control of mycobacteriosis, in this, study zebrafish were used for the identification and characterization of bacterial microbiota α-Gal content. Then, selected bacteria with high α-Gal content, *Aeromonas veronii* and *Pseudomonas entomophila*, were used as probiotics for the control of *Mycobacterium marinum* and the study of associated microbiota and immune-mediated mechanisms. The results showed that treatment with α-Gal and probiotics with high α-Gal content modified fish gut microbiota composition and activated protective mechanisms regulating immunity and metabolism.

## 2. Results and Discussion

### 2.1. Zebrafish Gut Microbiota Contains Potential Probiotic Bacteria with High α-Gal Content 

**Figure 1:**
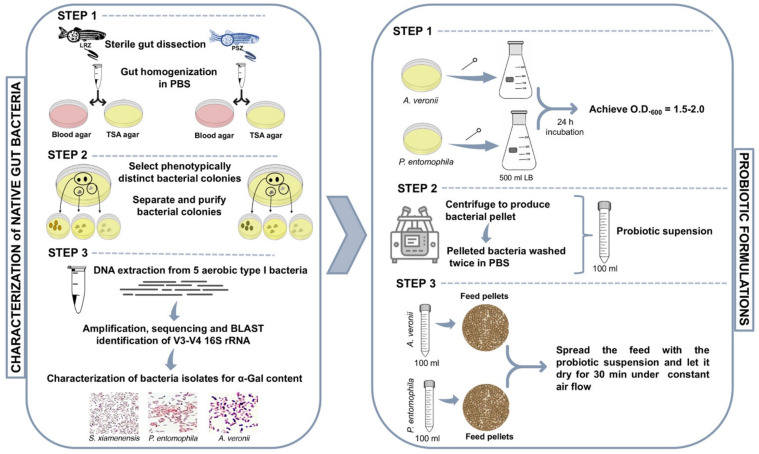
Methodology for the identification and characterization of zebrafish native gut potential probiotic bacteria. Adult female and male wild-type AB laboratory-reared zebrafish (LRZ) and pet-store zebrafish (PSZ) were used for analysis. Potential probiotic bacteria were isolated from the gut or gastrointestinal tract, and bacteria identified with high α-Gal content, namely *A. veronii* and *P. entomophila*, were used for probiotic formulations.

**Figure 2:**
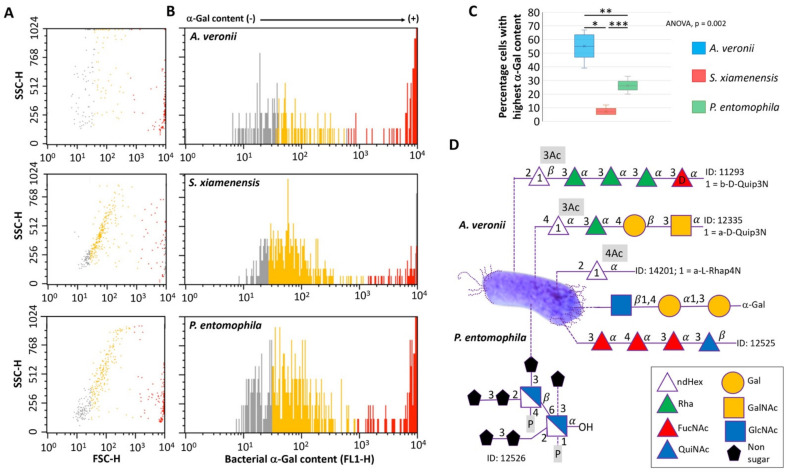
The carbohydrate structure and α-Gal content in potential probiotic bacteria. (**A**) Density plot representing bacteria that were gated by forward (FSC-H) and side (SSC-H) scatter. (**B**) Bacteria are represented in a histogram to evaluate the relative α-Gal levels (FL1-H). Cells were incubated with the α-Gal epitope monoclonal antibody M86. FITC-goat anti-mouse IgM-labeled antibodies were used as a secondary antibody. Samples were analyzed on a FAC-Scalibur flow cytometer equipped with CellQuest Pro software v.4. The viable cell population was gated according to forward-scatter (FSC-H) and side-scatter (SSC-H) parameters. (**C**) The percentage of viable cell population with highest α-Gal content (with mean fluorescence intensity >10^3^ FSC-H; red marks) was compared between different bacteria by one-way ANOVA test (*p* < 0.005) followed by post hoc Holm multiple comparisons (* *p* = 0.002, ** *p* = 0.02, *** *p* = 0.04, *n* = 5 biological replicates). (**D**) The bacterial carbohydrate structure for bacteria identified in the zebrafish microbiota with highest α-Gal content, namely *A. veronii* and *P. entomophila*, was characterized by using the Bacterial Carbohydrate Structure Database. The α-Gal was included as reported here in both bacteria. Compound IDs are shown. IUPAC condensed terms are disclosed in Materials and Methods.

A methodological approach was developed for the identification and characterization of zebrafish native gut potential probiotic bacteria (Figure 1). After incubation, each morphologically distinct colony (form, color, texture, elevation and margin) was encoded. From each sampling plate, two representatives of each colony were randomly selected and subcultured on a separate blood agar and isolated for downstream analyses. A total of two different bacterial community phenotypes were observed under the identification criteria, aerobic and anaerobic bacteria in both LRZ and PSZ groups (Table 1). Sanger sequencing and BLASTN searches of the V3/V4 16S rDNA of five bacterial isolates resulted in 98.4% to 99.8% identity to bacteria previously reported in zebrafish gut microbiota [37,38,39] (Table 2). Of them, entries with maximum identity corresponded to *P. entomophila* (99.8%), *S. xiamenensis* (99.8%) and *A. veronii* (99.3%) (Table 2). These bacteria were then selected for the characterization of α-Gal content and glycan structure (Figure 2A–D).

The results showed that all three bacteria have α-Gal on its surface (Figure 2A,B) with significantly highest relative levels (>10^3^ FSC-H) in *A. veronii* (55% cells) and *P. entomophila* (26% cells) when compared to *S. xiamenensis* (7%) (Figure 2C). As a reference, published data for *M. marinum* showed 3.2% cells with highest α-Gal content [24]. These results correlated with the reported carbohydrate structure in these bacteria, in which *A. veronii* but not *P. entomophila* contain galactose in addition to α-Gal in compound ID 12335 [-4)-a-D-Quip3NAc-(1–3)-a-L-Rhap-(1-4)-b-D-Galp-(1–3_-a-D-GalpNAc-(1-] [40] (Figure 2D).

Among fish-associated bacteria, *A. veronii* is found in fresh water in association with vertebrates and invertebrates with virulence factors such as enterotoxin, flagella and outer membrane proteins that affect fishes and other aquatic animals with high mortality rate and economic losses [41,42,43,44,45,46,47,48]. *Pseudomonas entomophila* is commonly found in insects and soil and is closely related to *P. putida* [49]. Together with *Aeromonas* spp., *Pseudomonas* spp. are among the most pathogenic Gram-negative bacteria in fish with resistance to multiple antibiotics commonly used in aquaculture [50,51]. However, *A. veronii* and *Pseudomonas* spp. are also symbionts with possible beneficial effects for the host [12,42,52].

### 2.2. Bacteria from Zebrafish Gut Microbiota with High Alpha-Gal Content Are Not Toxic

**Figure 3:**
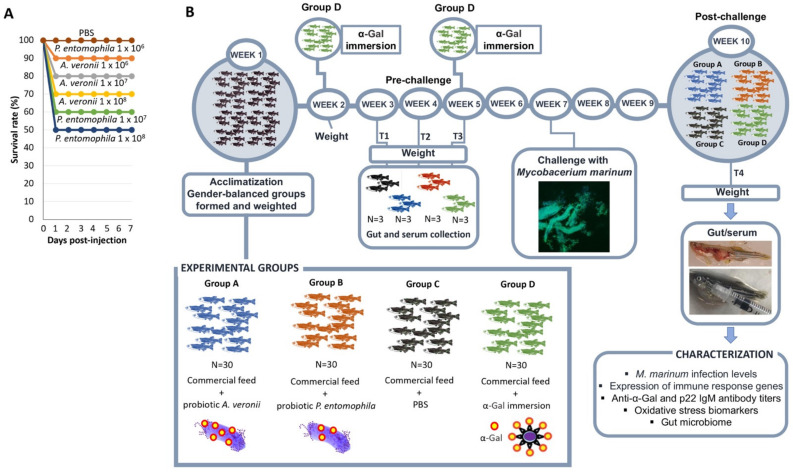
Evaluation of proposed probiotic bacteria in zebrafish. (**A**) Evaluation of bacterial biosafety. Ten fish per group were injected intraperitoneally with 1 × 10^6^, 1 × 10^7^ and 1 × 10^8^ CFU per fish for both *A. veronii* and *P. entomophila*, separately. Fish injected with PBS buffer were used as controls. Bacterial toxicity was evaluated by recording signs and symptoms of infection and mortality of the injected fish daily for 7 days. (**B**) Experimental design for protective response against *M. marinum*. The effect of immunization with zebrafish gut candidate probiotic bacteria was evaluated with α-Gal and PBS used as positive and negative controls, respectively. Thirty LRZ were randomly allocated to Group A, commercial diet with probiotic *A. veronii*; Group B, commercial diet with probiotic *P. entomophila*; Group C, commercial diet with PBS; and Group D, commercial diet with α-Gal immersion. Fish were weighted at the weeks 1–5 and 10 at the end of the experiment. Gut and sera were collected at weeks 3 (T1), 4 (T2) and 5 (T3) and at the end of the experiment (week 10; T4) and processed for gut and serum collection for analysis of antibody levels by ELISA, mycobacteria levels by RT-qPCR, expression of selected immune response gene markers by RT-qPCR, oxidative stress biomarkers and gut microbiome.

A basic requirement for probiotics is the safety in treated organisms. To assess this requirement, the toxicity of bacteria from the zebrafish gut microbiota with highest α-Gal content, namely *A. veronii* and *P. entomophila*, was evaluated by intraperitoneal injection of different bacterial doses. The results suggested low pathogenicity and toxicity of these potential probiotic bacteria even at high doses of 1 × 10^6^ CFU per fish (100% and 90% survival rate for *P. entomophila* and *A. veronii*, respectively; Figure 3A). The only symptom observed in treated fish before dead was abnormal behavior pattern. These results support the use of these bacteria for probiotic treatments in fish. 

### 2.3. Bacteria from Zebrafish Gut Microbiota with High Alpha-Gal Content Protect Fish against Mycobacteriosis

Bacteria from the zebrafish gut microbiota with highest α-Gal content and nontoxic were used as probiotics in fish challenged with *M. marinum* for the characterization of protective immune and oxidative stress responses and gut microbiome (Figure 3B).

**Figure 4:**
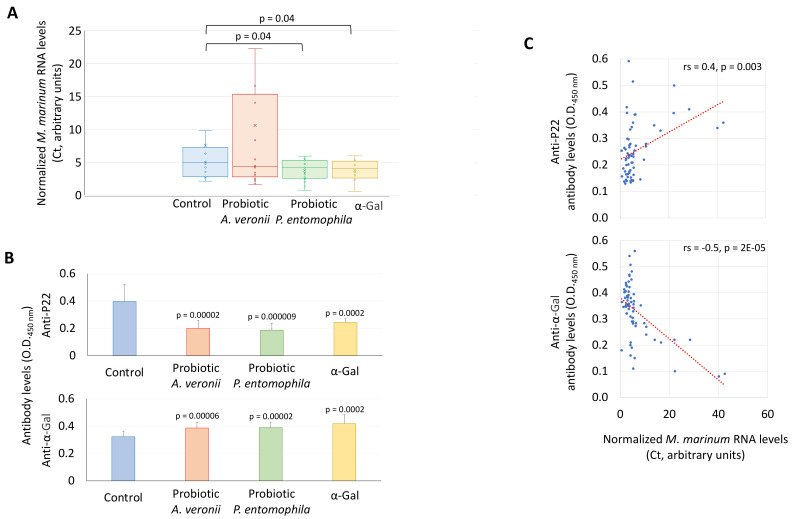
Effect of probiotic treatment and challenge with *M. marinum* on the zebrafish mycobacterial infection levels and antibody response. (**A**) *Mycobacterium* RNA levels were characterized by RT-qPCR in immunized and control PBS zebrafish challenged with *M. marinum*, normalized against *D. rerio gapdh*. The normalized Ct values were compared between treated and negative PBS control groups by Student’s *t*-test with unequal variance (*p* < 0.05; *n* = 10–17/group). (**B**) Anti-α-Gal and P22 IgM antibody titers were characterized by ELISA in immunized and control PBS zebrafish challenged with *M. marinum*. The o.d. at 450 nm (mean of the duplicate well values of o.d. P22 or α-Gal–o.d. PBS control plus standard deviation, SD) were compared between treated and negative PBS control groups at T4 by Student’s *t*-test with unequal variance (*p* < 0.005; *n* = 12–20/group). (**C**) Spearman’s Rho correlation analysis between antibody titers and *M. marinum* infection RNA levels (*p* < 0.005).

**Figure 5:**
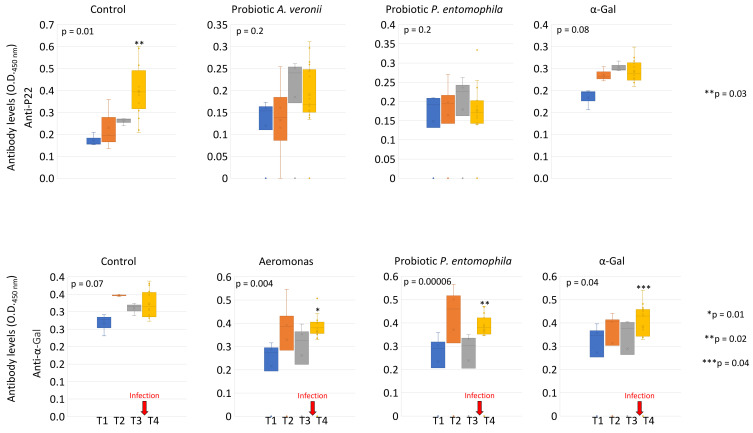
Anti-α-Gal and P22 IgM antibody titers in immunized and control PBS zebrafish challenged with *M. marinum*. Anti-α-Gal and P22 IgM antibody titers were characterized by ELISA. The o.d. at 450 nm (mean of the duplicate well values of o.d. P22 or α-Gal – o.d. PBS control) was compared between different time points (T1 to T4) by one-way ANOVA test (*p* < 0.05), followed by post hoc Holm multiple comparisons between T1 and T4 (* *p* < 0.01, ** *p* < 0.04, *** *p* < 0.05, *n* = 3–20/group). The time of infection challenge with *M. marinum* is shown with red arrows.

The effect of probiotic treatment and challenge with *M. marinum* was characterized on the zebrafish mycobacterial infection levels and antibody response (Figure 4A–C and Figure 5). High animal-to-animal variations in the *M. marinum* infection determined by mycobacteria RNA levels were observed in the group treated with the *A. veronii* probiotic (Figure 4A). Consequently, a significant difference in mycobacterial infection when compared to control fish was observed only in groups treated with the *P. entomophila* probiotic (44% decrease) and α-Gal (38% decrease) (Figure 4A). However, the IgM antibody levels against *Mycobacterium* P22 and α-Gal were significantly lower and higher in all probiotics or α-Gal treated groups when compared to controls, respectively (Figure 4B). Accordingly, anti-P22 antibody titers significantly increased from T1 (before *M. marinum* infection) to T4 (after infection) only in the control group while anti-α-Gal antibody levels increased only in treated groups (Figure 5). These results suggested that the previously demonstrated protective antibody response to α-Gal [23,24] increased in response to probiotics and α-Gal treatments, which translated into lower anti-P22 antibody levels likely reflecting reduction in mycobacterial infection. In support to this finding, a correlation analysis was conducted between antibody titers and *M. marinum* infection RNA levels to show a significant positive and negative correlation for anti-P22 and anti-α-Gal antibody titers, respectively (Figure 4C). The results of this trial supported that *A. veronii* and *P. entomophila* may be used as probiotics against fish mycobacteriosis.

### 2.4. Treatment with Probiotic Bacteria with High α-Gal Content Induce the Expression of Immune Response and Nutrient Metabolism Genes

**Figure 6:**
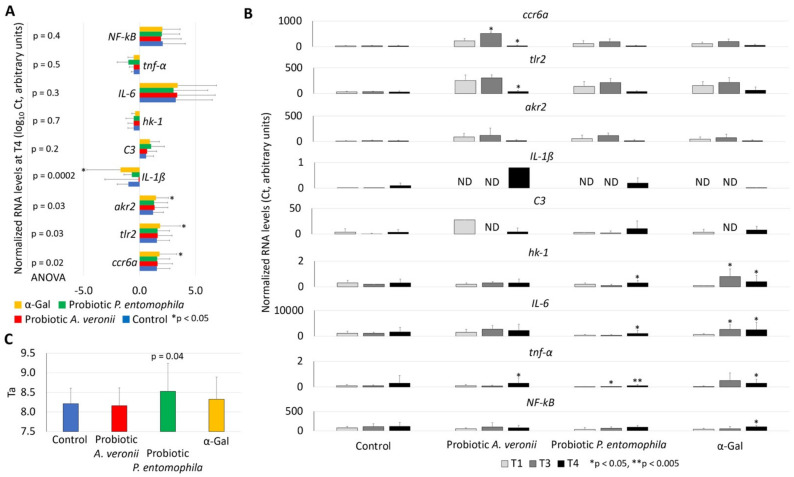
Expression of zebrafish immune-response genes in response to α-Gal and probiotic bacteria. The RT-qPCR was performed for the analysis of gene mRNA levels, using specific primers and conditions (Table 3). (**A**) The RNA normalized Ct values were compared between groups at T4 (end of the trial) by one-way ANOVA test followed by post hoc Holm multiple comparisons (https://astatsa.com/OneWay_Anova_with_TukeyHSD/; * *p* < 0.05, *n* = 3–20/group). (**B**) The RNA normalized Ct values were compared between T1 and T3/T4 by Student’s *t*-test with unequal variance (* *p* < 0.05, ** *p* < 0.005; *n* = 12–20/group). Abbreviation: ND, not detected. High-resolution graphs are shown in Supplementary Materials Figure S1. (**C**) Antioxidant capacity in serum (Ta) was determined by using the potassium permanganate method and Ta values were compared between treated and control groups at T4 by Student’s *t*-test with unequal variance (*p* < 0.05; *n* = 14–20/group). Data are shown as mean + SD.

For the characterization of probiotic-induced mechanisms, immune response (*ccr6a*, *tlr2*, *akr2*, *IL-1ß*, *C3*, *IL-6*, *tnf-α* and *NF-kB*) and nutrient metabolism (*hk-1*) genes were selected, as they were previously shown to be involved in zebrafish immune protective mechanisms and response to immunization with α-Gal and probiotics [16,24,32,53] (Figure 6A,B). The expression of selected genes was characterized in the gut involved in both innate and adaptive fish immunity [35,53,54]. The effect of treatment with probiotics or α-Gal and *M. marinum* infection at the end of the trial (T4) corroborated previous results in α-Gal-immunized zebrafish [24] with upregulation of *ccr6a, tlr2*, *ak2* and *IL-1ß* (Figure 6A). The characterization of the effect of probiotics/α-Gal treatments before (T1/T3) and after (T4) infection with *M. marinum* showed upregulation of *hk-1* and *IL-6* in response to α-Gal treatment and upregulation of *hk-1*, *IL-6*, *tnf-α* and *NF-kB* in response to α-Gal treatment and *M. marinum* infection (Figure 6B; Supplementary Materials Figure S1). The treatment with probiotic *A. veronii* resulted in the upregulation of *ccr6a* and *tnf-α* before and after mycobacterial infection, respectively (Figure 6B; Supplementary Materials Figure S1). However, the *ccr6a* and *tlr2* mRNA levels decreased after treatment with probiotic *A. veronii* and *M. marinum* infection (Figure 6B; Supplementary Materials Figure S1). The treatment with *P. entomophila* upregulated *tnf-α* before infection and *tnf-α*, *IL-6* and *hk-1* after infection (Figure 6B). Gene expression levels did not vary in control zebrafish (Figure 6B; Supplementary Materials Figure S1), thus supporting those changes in gene mRNA were not in response to mycobacterial infection only.

### 2.5. Treatment with Probiotic Bacteria with High α-Gal Content Reduces Oxidative Stress in Fish

The serum total antioxidant capacity (Ta) was used to evaluate the effect of probiotics with high α-Gal content on fish oxidative stress (Figure 6C). The results showed a significant increase in Ta in fish treated with probiotic *P. entomophila* (Ta = 8.528 ± 0.711 vs. 8.210 ± 0.396 in control group). This Ta value is high when using human sera from young individuals as a reference [55] and supports an effect of probiotic *P. entomophila* on reducing the oxidative stress in treated fish. Probiotic treatments have resulted in increased serum total antioxidant capacity to facilitate prevention of oxidative stress that causes cellular damage and affects immune response in fish [56,57,58,59]. Furthermore, modulation of oxidative defenses has been correlated with protection against mycobacteriosis in fish [60,61].

### 2.6. Microbiota Composition Varies in Response to Treatment with Probiotic Bacteria and α-Gal

Immune training by fish gut microbiota is a core mechanism for the activation of protective responses against pathogen infection [31]. In humans, the composition of gut microbiota and microbiome driven immunomodulation affect protection against tuberculosis [62]. However, in fish these mechanisms are poorly understood.

Our study characterized the zebrafish gut microbiota to explore the effect of *A. veronii* and *P. entomophila* probiotics and α-Gal treatments on microbial populations and the immune response to *M. marinum*. Following *16S rRNA* gene sequencing and filtering a total of 8922 amplicon sequence variants (ASVs) were assigned and distributed into 39 phyla, 93 classes, 205 orders, 311 families and 646 genera (Supplementary Materials File S1: Data S1), using the DADA2 algorithm. For further analysis, ASVs with low counts and those with prevalence lower than 0.01% were filtered to remove spurious ASVs in the bacterial dataset. The results showed that the zebrafish gut microbiota in all experimental groups and at each time point (pre-challenge and post-challenge) is dominated by members of the phylum Proteobacteria (genera *Aeromonas*, *Acinetobacter, Gemmobacter* and *Plesiomonas*) followed by Bacteroidota (genera *Cloacibacterium*), Firmicutes, Actinobacteria and Planctomycetota phyla (Supplementary Materials File S1: Figures S2–S4). These microbial composition trends have been previously reported in the zebrafish gut microbiota [37,63].

**Figure 7:**
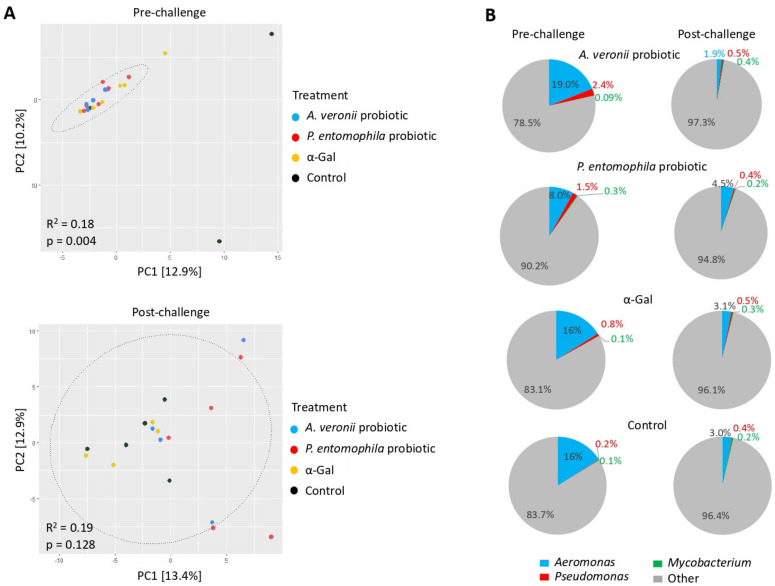
Zebrafish gut microbiota composition in response to probiotic and α-Gal treatments. (**A**) Principal component analysis of zebrafish gut microbiota grouped by treatment at pre-challenge and post-challenge stages. PCA ordination is based on the Bray–Curtis dissimilarity calculated with randomly rarefied data with no replacement applied to the centered-log transformed clr counts. The percentage of variation is explained by the principal components in the axis, PC1 and PC2. Ellipses indicate 95% confidence intervals. Each point represents one sample, and colors represent treatments/control groups. The closer the points are to one another, the more similar the microbiome composition of the samples are and vice versa. Adonis function in R software was used for PERMANOVA test to evaluate differences between groups. (**B**) Pie charts display the relative abundance of the genera *Aeromonas*, *Pseudomonas*, *Mycobacterium* and other found on each treatment group at pre-challenge and post-challenge stages. Relative abundance (%) of each genus was calculated from the ASVs raw counts obtained with DADA2 and normalized by total sum scaling.

To examine the dissimilarities in community composition between experimental groups at each time point (pre-challenge and post-challenge), beta diversity metric was assayed by using Bray–Curtis dissimilarity in univariable PERMANOVA models. The results showed significant differences between experimental groups (*A. veronii* probiotic treatment, *P. entomophila* probiotic treatment, and α-Gal) and controls at the pre-challenge stage (*p* = 0.004, R^2^ = 0.18), but not at the post-challenge stage (*p* = 0.128, R^2^ = 0.19) (Figure 7A). These results suggested that before *M. marinum* challenge the gut zebrafish microbial community differences observed might be attributed to the effect of treatments (*A. veronii* probiotic, *P. entomophila* probiotic and α-Gal) (Figure 7A). However, the challenge with *M. marinum* likely resulted in the disturbance of gut microbiota in all experimental groups (Figure 7A). From the taxonomic assignments, we observed that the genera *Aeromonas*, *Pseudomonas* and *Mycobacterium* are present in all experimental groups at both pre-challenge and post-challenge time points (Figure 7B), thus providing evidence of their ubiquity within the zebrafish microbial community. At the pre-challenge stage, the relative abundance of the genus *Aeromonas* in the *A. veronii* probiotic, α-Gal and control groups was higher than the genus *Pseudomonas* (Figure 7B). This finding could be associated with differences in the colonization rates and gut adaptation requirements for each bacterium and as the result of competitive microbial interactions [63,64]. In response to the *M. marinum* challenge, a decrease in the relative abundance of the genera *Aeromonas* and *Pseudomonas* occurred throughout all the experimental groups (Figure 7B).

**Figure 8:**
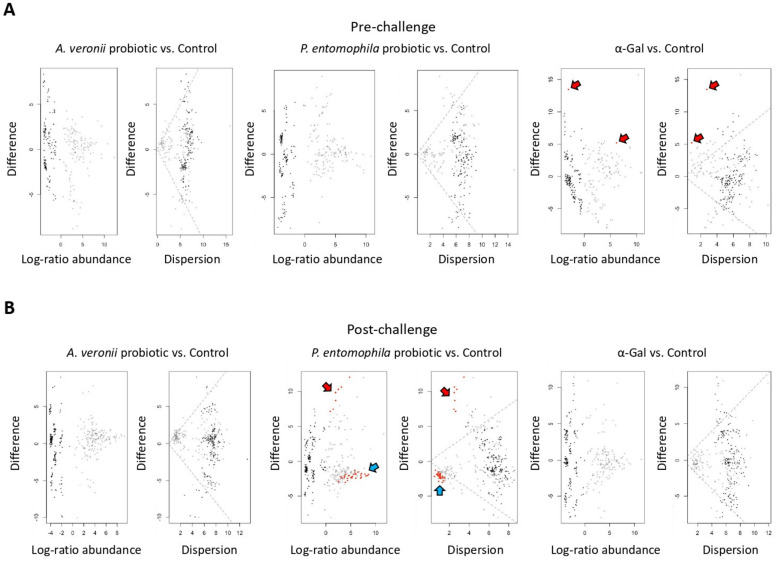
Differential abundance of bacterial taxa in zebrafish gut microbiota. (**A**) Taxa differential abundance of each treatment group vs. control at pre-challenge stage. (**B**) Taxa differential abundance of each treatment group vs. control at post-challenge stage. Taxa differential abundance was calculated with ALDEx2 and summarized in the effects size plots. The left MA plots (log-ratio abundance) show the relationship between abundance (log-ratio abundance is the clr value of each feature) on the *x*-axis and difference on the *y*-axis. The right plot (dispersion) is an effect plot that shows the relationship between difference and dispersion through the expected value of the log-difference between groups on the *y*-axis and the maximum within-group dispersion on the *x*-axis. In both plots, each point represents an individual ASV from the dataset at genus level. Taxa that are not significant are represented by gray or black points. Taxa that are statistically significant are represented by red points (Welch’s test, *p* < 0.05). Points marked with red arrows are more abundant in α-Gal treatment samples at pre-challenge stage or in *P. entomophila* probiotic treatment samples at post-challenge stage when compared to controls. Points marked with blue arrows are more abundant in control than in *P. entomophila* probiotic treatment samples at post-challenge stage.

Then, we further explored whether the gut microbiota differences observed between the experimental groups are induced by changes in the abundance of specific taxa. The differentially abundant taxa of the zebrafish gut microbiota are displayed in the effect size plots shown in Figure 8A,B for *A. veronii* and *P. entomophila* probiotics and α-Gal treatment groups in comparison to controls at pre-challenge and post-challenge stages. The effect size plots showed the presence of differential taxa at both pre-challenge and post-challenge stages (Figure 8A,B). We filtered the list of significantly different taxa found in the effect size plots of both groups generated with ALDEx2 to show only those taxa for which the expected Benjamini–Hochberg *p*-value was less than 0.05 (Table 3). From these results, we can conclude that, at pre-challenge stage, the *A. veronii* and *P. entomophila* probiotic treatments do not affect to the whole structure of the zebrafish gut microbiota, as no differential abundant taxa were found in in those groups. Based on our identification of bacteria with high α-Gal content in Zebrafish gut microbiota (Figure 2 and Table 2), this is an expected result, as *A. veronii* and *P. entomophila* are natural resident bacteria of zebrafish (Table 2) and other fish species [12]. Therefore, no disturbance was observed at the pre-challenge stage for treatments with these probiotic bacteria. In contrast, at the pre-challenge stage the comparison of α-Gal treatment and control groups resulted in few differential taxa (Figure 8A), a result that supports a role for α-Gal glycan in shaping the microbiota composition [25,65,66]. However, at the post-challenge stage differentially abundant taxa were observed only in the *P. entomophila* probiotic treatment when compared to the control group (Figure 8B). These results suggested a change in the gut microbial community composition in zebrafish treated with *P. entomophila* probiotic and infected with *M. marinum*. Nevertheless, whether these observed differential taxa were directly related to *M. marinum* infection by means of competition and/or activation of immune system pathways needs to be further explored.

**Figure 9:**
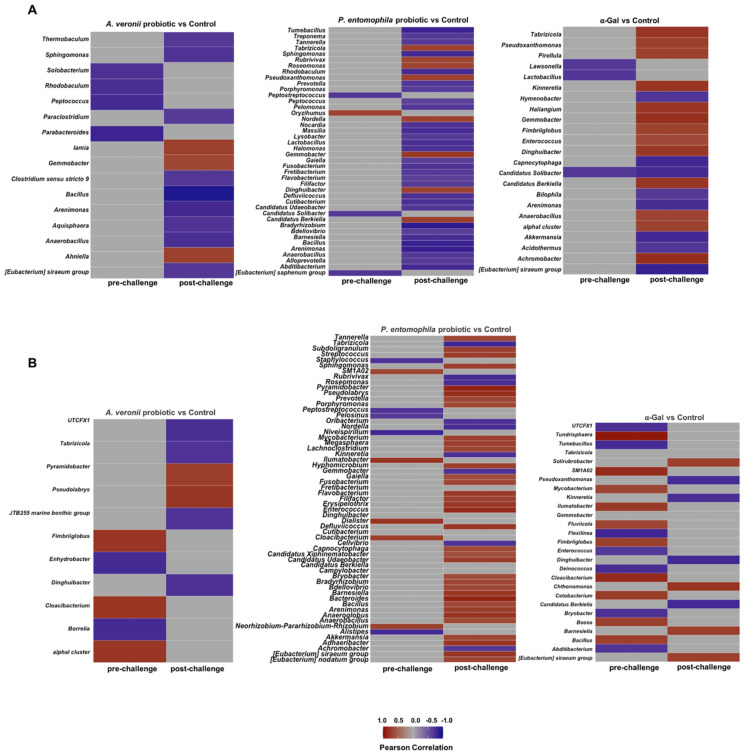
Correlation between zebrafish gut microbiota and antibody response. Heatmaps of the significantly correlated taxa with (**A**) anti-α-Gal and (**B**) anti-P22 IgM titers for each experimental group and at each stage (pre-challenge, post-challenge). Pearson correlations between zebrafish gut microbiota and anti-α-Gal and P22 IgM antibody titers were calculated with ALDEx2, using the aldex.corr function analysis, as implemented in R.

To characterize a possible association between the zebrafish gut microbiota and the IgM antibody response to α-Gal and *Mycobacterium* P22, a correlation analysis was performed between the abundance of bacterial taxa at genus level and antibody titers. We observed a pattern of significantly correlated taxa with anti-α-Gal IgM in all the experimental groups when compared at the pre-challenge and post-challenge time points, with a notable increase of significant taxa that correlate positively at the post-challenge time point in the α-Gal treatment group (Figure 9A). It has been demonstrated that certain bacteria from the zebrafish gut microbiota contain α-Gal on its surface (Table 2) [35], and thus the immunity induced in response to α-Gal treatment may negatively affect the zebrafish gut microbiota bacteria containing α-Gal [67,68]. In contrast, we did not observe a clear pattern of the significantly correlated taxa with anti-P22 IgM antibody titers (Figure 9B).

### 2.7. Mechanisms Mediating Protection against Mycobacteriosis by Probiotics with High α-Gal Content

**Figure 10:**
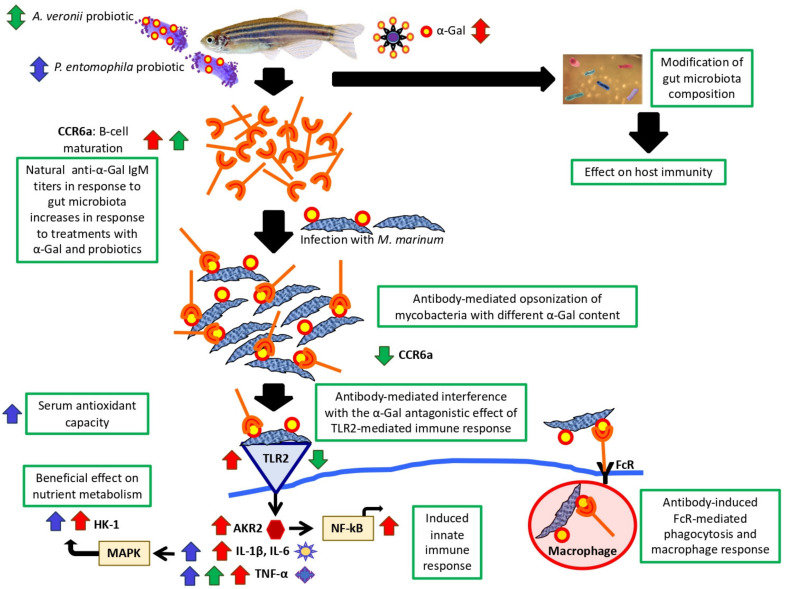
Protective mechanisms activated in response to α-Gal and probiotics with high α-Gal content. Mechanisms regulating immunity and metabolism induced by α-Gal and probiotic bacteria with high α-Gal content included modification of gut microbiota composition, B-cell maturation, anti-α-Gal antibodies-mediated control of mycobacteria, induced innate immune responses, beneficial effects on nutrient metabolism and reduced oxidative stress. Probiotics activated different mechanisms associated with the response to α-Gal.

Based on the results of this study together with previous findings in fish immunized with α-Gal or heat-inactivated *M. bovis* and protected against mycobacteriosis [24,32,33], we proposed mechanisms regulating immunity and metabolism induced by probiotic bacteria with high α-Gal content (Figure 10). These mechanisms included B-cell maturation, anti-α-Gal antibodies-mediated control of mycobacteria, induced innate immune responses and beneficial effects on nutrient metabolism and oxidative stress. Additionally, in the zebrafish model, the results suggested a role of immune system pathways in response to probiotics and α-Gal that are related to the microbiota composition [69,70,71].

Mycobacteria contain α-Gal on their surface, and zebrafish, similar to humans, evolved as α-Gal negative and produce natural anti-α-Gal antibodies in response to bacteria in the gut microbiota with this modification [24,35]. Therefore, the increase in the antibody levels to α-Gal in response to probiotics support a protective mechanism by antibody-mediated opsonization of mycobacteria through interactions with surface-exposed antigens and promotion of Fc-receptor (FcR)-mediated phagocytosis [24,72]. In accordance with these results, the expression of the CCR6a beta chemokine receptor coding gene that is implicated in B-lineage maturation and antigen-driven B-cell differentiation and humoral immunity [73], was upregulated in response to α-Gal and probiotic *A. veronii* to promote the production of anti-α-Gal antibodies. To interfere with protective responses induced by treatment with probiotic *A. veronii*, *M. marinum* infection downregulated the expression of *ccr6* and *tlr2*. 

The upregulation of proinflammatory cytokines (ILs and TNF-α) through the TLR/NF-kB-AKR innate immune pathway has been implicated in the α-Gal-induced protective mechanism to mycobacterial infection [24,72,74,75,76,77]. The activation of macrophages by anti-α-Gal antibodies increases TNF-α secretion which may promotes macrophage recruitment to the infection site with a role during the initial and long-term control of tuberculosis [24,78]. Additionally, α-Gal on mycobacterial membrane may be similar to glycolipids that antagonize TLR2-mediated response to inhibit NF-kB/AKR activation and subsequent cytokine production, a process which may be interfered by the anti-α-Gal antibodies [24,77]. 

In fish as in other organisms the enzyme HK-1 has a role in glycolysis [79]. Higher expression levels of *hk-1* in response to treatment with α-Gal and probiotic *P. entomophila* suggested a beneficial effect of α-Gal-containing probiotic bacteria on nutrient metabolism. Similar results have been reported in fish treated with xylanase-producing probiotics [16,34,80]. Additionally, proinflammatory cytokines such as TNF-α and IL-6, upregulated here by α-Gal and probiotic *P. entomophila*, have been implicated in the regulation of HK-1 through the mitogen-activated protein kinase (MAPK) pathway [81].

Probiotic *A. veronii* and *P. entomophila* specific signatures showed differences associated with these treatments (Figure 10). *A. veronii* with higher α-Gal content was the only probiotic inducing B-cell maturation, which was reverted by *M. marinum* infection, a finding that together with *tlr2* downregulation may explain the absence of significant differences in mycobacterial infection levels in these fish (Figure 4A). In contrast, probiotic *P. entomophila* was the only upregulating IL-6 resulting in the MAPK-mediated induction of HK-1-associated beneficial effect on nutrient metabolism. Probiotic *P. entomophila* was also the only treatment resulting in an increase of serum total antioxidant capacity, which facilitates immune response by preventing the oxidative stress in these fish. Finally, both probiotic bacteria induced innate immune response trough TNF-α upregulation. Other mechanisms associated with TLR2, AKR2, NF-kB and IL-1ß were regulated only in response to α-Gal and induced modifications in gut microbiota composition may enhance the protective response to infection.

## 3. Materials and Methods

### 3.1. Zebrafish

Wild-type adult (6–8 months old) AB female and male laboratory-reared zebrafish (LRZ) were kindly provided by Juan Galcerán Sáez from the Instituto de Neurociencias (IN-CSIC-UMH, Sant Joan d’Alacant, Alicante, Spain). These zebrafish were certified by Biosait Europe S.L. (Barcelona, Spain; https://biosait.com) as free of major fish pathogens, such as *Mycobacterium* spp., *Pseudoloma neurophilia*, *Pseudocapillaria tomentosa* and zebrafish retroviruses. Pet-store zebrafish (PSZ) female and male adults (6–8 months old) were purchased from a pet store in Ciudad Real (Spain) and transported to the microbiology laboratory installations at the IREC for immediate processing. The zebrafish were maintained in a flow-through water system at 27 °C, with a light/dark cycle of 14 h/10 h, and fed twice daily with dry fish feed (Premium food tropical fish, DAPC, Valladolid, Spain). Experiments were conducted in strict accordance with the recommendations of the European Guide for the Care and Use of Laboratory Animals. Animals were housed and experiments conducted at the experimental facility (IREC, Ciudad Real, Spain) with the approval and supervision of the Ethics Committee on Animal Experimentation of the University of Castilla La Mancha (PR-2018-06-13) and the Counseling of Agriculture, Environment, and Rural Development of Castilla La Mancha (ES130340000218).

### 3.2. Sampling and Bacterial Culture from Zebrafish Gut Microbiota

Potential probiotic bacteria were isolated from the gut of LRZ and PSZ (*n* = 10 each) (Figure 1). The culturable microbiota was sampled as previously described [38]. The ventral belly surface of freshly euthanized fish was opened with sterilized microsurgical blade and forceps under a light source. The intestinal system was transferred to 1.5 mL tubes containing 200 μL sterile PBS. The intestines were homogenized with a motorized pestle and disposable plastic loops were used to streak four serial dilutions on 5% chicken blood agar (Rockland antibodies and assays, Rockland Immunochemicals, Inc., Limerick, PA, USA) and TSA agar (Sigma-Aldrich, St. Louis, MO, USA) bacteriological plates for isolation of aerobic and anaerobic bacteria, respectively. The plates were incubated at 28 ºC and followed by inspections every day for up to 1 week. After incubation, each morphologically distinct colony (form, color, texture, elevation and margin) was encoded. From each sampling plate, two representatives of each colony were randomly selected, subcultured on separate blood agar and isolated for downstream analyses. A total of 5 phenotypes of different bacterial colonies were isolated in both LRZ and PSZ groups and classified (Table 1).

### 3.3. Bacterial DNA Extraction and 16S rRNA Gene Amplification and Sequencing

Genomic DNA was extracted from 5 different aerobic bacterial type I colony isolates (Figure 1 and Table 1) from LRZ and PSZ, using the direct boiling method [82]. The amplification of the 16S rRNA gene V3/V4 regions was carried out by PCR, using the primers 16S-341F: 5′-TCGTCGGCAGCGTCAGATGTGTATAAGAGACAGCCTACGGGNGGCWGCAG-3’ and 16S_805R: 5′-GTCTCGTGGGCTCGGAGATGTGTATAAGAGACAGGACTACHVGGGTATCTAATC-3’ in a final volume 25 µL (2 µL of DNA template (20 ng), 16 µL H_2_O, 0.5 µL of dNTPs (10 nM), 2.0 µL of MgCl2 (25 mM), 0.1 μL AmpliTaq Gold DNA polymerase (Life Technologies, UK), 1 µL of each primer (10 nM) and 2.5 µL PCR Gold Buffer (Life Technologies, Carlsbad, CA, USA). An initial denaturation step at 95 °C for 10 min was followed by 35 cycles of pre-amplification at 94 °C for 30 s, 55 °C for 30 s and 72 °C for 30 s, followed by a final elongation step at 72 °C for 10 min. All PCR products were purified by using the ExoSap-IT PCR Product Clean-Up kit (Applied Biosystems, Foster City, CA, USA) following the manufacturer’s instructions and Sanger sequenced, using the ABI PRISM^®^ 3730 platform (Applied Biosystems) at the Genomic Unit (Campus Moncloa, University Complutense of Madrid, Madrid, Spain; https://www.ucm.es/english/genomics-and-proteomics). All the V3/V4 16S rRNA gene sequences were edited by using SnapGene software (https://www.snapgene.com, accessed on 30 June 2020) and the maximum identities were searched by using the GenBank DNA sequence database and the Nucleotide Basic Local Alignment Search Tool (BLASTN; https://blast.ncbi.nlm.nih.gov/Blast.cgi, accessed on 30 June 2020) (Table 2). 

### 3.4. Analysis of Bacterial α-Gal Content 

The analysis of α-Gal content was conducted in selected aerobic type I bacteria with maximum 16S rRNA gene sequence identity (99.3–99.8%; Table 2) (Figure 1). The *P. entomophila* (type strain, DSM 28517), *S. xiamenensis* (type strain, DSM 22215) and *A. veronii* (type strain, DSM 7386) were obtained from the German Collection of Microorganisms and Cell Culture (DSMZ Leibniz Institute, Braunschweig, Germany; https://www.dsmz.de). The flow cytometry analysis of bacterial α-Gal content was conducted as previously described [24,35]. Bacteria were washed in PBS, fixed and permeabilized with the Intracell fixation and permeabilization kit (Immunostep, Salamanca, Spain) following manufacturer’s recommendations. The cells were incubated with 3% human serum albumin (HSA, Sigma-Aldrich) in PBS, for 1 h, at room temperature (RT). Then, cells were incubated for 14 h at 4 °C with the α-Gal epitope monoclonal antibody (M86, Enzo Life Sciences, Farmingdale, NY, USA) diluted 1:50 in 3% HSA/PBS. FITC-goat anti-mouse IgM (Abcam, Cambridge, UK) labelled antibody (diluted 1/200 in 3% HSA/PBS) was used as a secondary antibody and incubated for 1 h, at RT. Samples were analyzed on a FAC-Scalibur flow cytometer equipped with CellQuest Pro software v.4 (BD Bio-Sciences, Madrid, Spain). The viable cell population was gated according to forward-scatter (FSC-H) and side-scatter (SSC-H) parameters. The viable cell population was gated according to forward-scatter (FSC-H) and side-scatter (SSC-H) parameters. The percentage of viable cell population with highest α-Gal content (mean fluorescence intensity >10^3^ FSC-H) was compared between different bacteria by one-way ANOVA test (*p* < 0.005) followed by post hoc Holm multiple comparisons (https://astatsa.com/OneWay_Anova_with_TukeyHSD/; *p* < 0.05, *n* = 5 biological replicates).

### 3.5. Bacterial Carbohydrate Structure

The bacterial carbohydrate structure for bacteria identified in the zebrafish microbiota with highest α-Gal content, namely *A. veronii* and *P. entomophila*, was characterized by using the Bacterial Carbohydrate Structure Database (http://csdb.glycoscience.ru/bacterial/main.html) [83,84,85,86]. Symbol nomenclature for glycans is disclosed at the database (http://csdb.glycoscience.ru/database/index.html?help=eog). The International Union of Pure and Applied Chemistry (IUPAC; https://iupac.org) condensed terms for the glycan structure are ndHex (Deoxy-hexose; http://www.monosaccharidedb.org/display_monosaccharide.action?name=deoxy-HEX), Rha (L-Rhamnose; http://www.monosaccharidedb.org/display_monosaccharide.action?name=?LRhap), FucNAc (*N*-acetyl-L-fucosamine; http://www.monosaccharidedb.org/display_monosaccharide.action?name=LFucpNAc), QuiNAc (*N*-acetyl-D-quinovasomine; http://www.monosaccharidedb.org/display_monosaccharide.action?name=DQuipNAc), Gal (D-galactose; http://www.monosaccharidedb.org/display_monosaccharide.action?name=?DGalp), GalNAc (*N*-acetyl-D-galactosamine; http://www.monosaccharidedb.org/display_monosaccharide.action?name=DGalpNAc) and GlcNAc (*N*-acetylglucosamine; https://pubchem.ncbi.nlm.nih.gov/compound/*N*-Acetyl-D-Glucosamine). All databases were accessed in 28 February 2021.

### 3.6. Probiotic Bacteria

Bacteria identified in the zebrafish microbiota with highest α-Gal content, *A. veronii* (type strain, DSM 7386) and *P. entomophila* (type strain, DSM 28517) were used for probiotoic preparation (Figure 1). The strains were inoculated on Luria broth (LB) agar plates for pure culture by using bacterial incubator to provide appropriate temperature to bacterial growth at 37 ºC for *A. veronii* and 28 °C for *P. entomophila* for 24 h. The strains cultured on LB agar plates were stored at 4 °C for use. Bacteria were cultured on LB agar plates repeatedly every 1 to 2 days to keep them viable. Moreover, the cultures were also stored in LB liquid medium containing sterile 50% glycerol at −80 °C for long-stem storage.

### 3.7. Toxicity Assessment of A. veronii and P. entomophila

Toxicity of probiotic bacteria was assessed as previously described [16]. Adult female and male PSZ (6–8 months old; *n* = 80) with an average weight of 266 ± 59 mg were acclimatized for 7 days as described above (Section 3.1). A total of 10 fish per group were injected intraperitoneally with 10 μL of the diluted bacterial solution of 1 × 10^6^, 1 × 10^7^ and 1 × 10^8^ CFU per fish for both *A. veronii* and *P. entomophila*, separately. Fish injected with PBS buffer were used as controls. Bacteria were cultured in LB broth for 24 h, at 37 and 28 °C for *A. veronii* and *P. entomophila*, respectively, and centrifuged at 4600× *g* for 20 min at 4 °C. The cell pellets were then suspended in an appropriate volume of PBS. Bacterial toxicity was evaluated by recording signs and symptoms of infection and mortality of the injected fish daily for 7 days.

### 3.8. Probiotic Formulation and Feed Administration

The probiotic formulation was prepared by using the coating and drying procedure [87] with some modifications (Figure 1). The probiotic suspension was prepared in 500 mL of fresh LB to grow *A. veronii* and *P. entomophila* for 24 h at 37 and 28 °C, respectively, and achieving an o.d. 600 of 1.5–2.0. Then, cultures were centrifuged at 4600× *g* for 20 min at 4 °C to produce the bacterial pellet. Pelleted bacteria were then washed twice in 1 mL sterile PBS and approximately 2 g of cell mass were diluted in 100 mL of sterile PBS to make the final probiotic suspension. The probiotic suspension was prepared freshly every week during the duration of the experiment. The probiotic suspension prepared for *A. veronii* and *P. entomophila* was manually spread in petri dishes to coat the feed and let it dry for 30 min under constant airflow. Finally, the probiotic-treated groups received a commercial staple food consisting of soft granules with 4% insect meal (Sera Vipagran Nature, D52528, Heinsberg, Germany) containing the probiotic bacteria tested at a final concentration of 10^8^ CFU/g. The probiotic bacterium was mixed into the diet before feeding and prepared freshly every day during the duration of the experiment. The viability of the probiotic suspension was monitored in the probiotic diet by plate count from 1 g of the probiotic suspension coated feed incubated for 5 min in 9 mL of sterile PBS, gently homogenized and serial dilutions cultured for 24 h on LB agar at 37 °C or 28 °C for *A. veronii* and *P. entomophila* probiotic suspension, respectively. All fish received a quantity of food ranging from 1.5% to 2% of their body weight per day during the experiment.

### 3.9. Zebrafish Treatment with Probiotics and Challenge with M. marinum

The experiment was designed to evaluate the effect of treatment with zebrafish gut candidate probiotic bacteria. Bovine serum albumin (BSA) coated with α-Gal (α-Gal; Dextra, Shinfield, UK) and PBS were used as positive and negative controls, respectively (Figure 3). Thirty LRZ were randomly allocated to different experimental groups with a similar number of adult females and males (Group A: commercial feed with probiotic *A. veronii*, Group B: commercial feed with probiotic *P. entomophila*, Group C: commercial feed with PBS, Group D: commercial feed with α-Gal immersion). For α-Gal treatment, fish were immunized by immersion n 200 mL of water from the fish tanks where 5 µg of α-Gal was added per fish for 30 min at weeks 2 and 5 (Figure 3). PBS was added to the commercial diet at a proportion of 500 µL per gram feed. Fish were weighted at the weeks 1–5 and 10 at the end of the experiment. Gut and sera were collected at weeks 3 (T1), 4 (T2) and 5 (T3) (3 fish per group) and at the end of the experiment (week 10; T4). The *M. marinum* Aronson (ATCC 927) was cultured at 29 °C in 7H9 broth enriched with Middlebrook ADC (Becton Dickinson) and prepared for infection as previously described [24,27,32,33]. To verify the bacterial dose, *M. marinum* samples were diluted and plated on 7H10 agar enriched with Middelbrook OADC (Becton Dickinson) for counting bacterial colonies. Fish were mucosally infected at week 7 with a dose equivalent to 48±7 cfu of *M. marinum* per animal causing a chronic tuberculosis-like disease in zebrafish [24,33]. At week 10, fish were euthanized with immersion in 0.04% MS-222 and processed for gut and serum collection for analysis of antibody levels by ELISA, mycobacteria levels by RT-qPCR, expression of selected immune response gene markers by RT-qPCR, oxidative stress biomarkers and gut microbiome. The zebrafish had a weight of 614 ± 259 and 694 ± 152 mg (Group C: PBS control), 643 ± 269 and 540 ± 181 mg (Group A: probiotic *A. veronii*), 681 ± 300 and 771 ± 299 mg (Group B: probiotic *P. entomophila*), and 463 ± 215 and 593 ± 336 mg (Group D: α-Gal) at the beginning and end of the experiment, respectively.

### 3.10. Characterization of M. marinum RNA Levels by RT-qPCR

Total RNA was extracted from zebrafish gut samples by using the AllPrep DNA/RNA/Protein kit (Qiagen, Hilden, Germany). The *M. marinum* RNA levels were determined by real-time reverse transcription polymerase chain reaction (RT-qPCR), using the iTaq^TM^ Universal SYBR Green One-Step Kit (BioRad, CA, USA) in the CFX96^TM^ Real-Time System (BioRad) thermocycler following manufacturer’s recommendations with specific primers and conditions for *M. marinum heat-shock protein 65* gene (*hsp65*; Genebank accession number: AF547855.1) [88] (hsp65Forward-F: 5′-CAACCCGCTCGGTCTGAA-3′, hsp65Reverse-R: 5′-CGACCTCTTTGGCCGACTT-3′, annealing at 59 °C for 30 s). A dissociation curve was run at the end of the reactions to ensure that only one amplicon was formed and that the amplicon denatured consistently in the same temperature range for every sample [89]. The RNA cycle threshold (Ct) values were normalized against *D. rerio glyceraldehyde-3-phosphate dehydrogenase* gene (*gadph*; NM_001115114.1) (gadphF: 5′-CGTGGTGCCAGTCAGAACAT-3′, gadphR: 5′-AGTCAGTGGACACAACCTGG-3′, annealing at 56 °C for 30 s), using the genNormddCT method [90]. Cross-reactivity of the primers with probiotic bacteria was discarded by in silico *hsp65* sequence alignment and RT-PCR. The *M. marinum* RNA normalized Ct values were compared between treated and negative PBS control groups by Student’s *t*-test with unequal variance (*p* < 0.05; *n* = 10–17/group).

### 3.11. Characterization of Anti-α-Gal and P22 IgM Antibody Titers in Zebrafish

For ELISA, high absorption capacity polystyrene microtiter plates were coated with 100 ng per well of α-Gal or *M. bovis* P22, an immunopurified subcomplex of bovine purified protein derivative (bPPD) [91] in carbonate–bicarbonate buffer (Sigma-Aldrich). After an overnight incubation at 4 °C, coated plates were washed one time with 300 µL/well PBS/0.05% Tween 20 (PBST; Sigma-Aldrich), and then blocked with 100 µL/well of 1% HSA (Sigma-Aldrich) at RT for 1 h. A dilution curve with 1:10, 1:100 and 1:1000 fish serum peritoneal fluid samples was performed and then diluted (1:10, *v/v*) in blocking solution and 100 µL/well were added into the wells of the antigen-coated plates and incubated for 1.5 h at 37 °C. Plates were washed three times with PBST and 100 µL/well of species-specific rabbit anti-zebrafish IgM antibodies diluted (1:1000, *v/v*) in blocking solution were added and incubated at RT for 1 h. Plates were washed three times with 300 µL/well of PBST. A goat anti-rabbit IgG-peroxidase conjugate (Sigma-Aldrich) was added diluted 1:3000 in blocking solution and incubated at RT for 1 h. After four washes with 100 µL/well of PBST, 100 µL/well of 3,3′,5,5′-tetramethylbenzidine (TMB) one solution (Promega, Madrid, Spain) were added and incubated for 15 min at RT. Finally, the reaction was stopped with 50 µL/well of 2 N H_2_SO_4_, and the o.d. was measured in a spectrophotometer at 450 nm. The o.d. at 450 nm (mean of the duplicate well values of o.d. P22 or α-Gal – o.d. PBS control) were compared between treated and negative PBS control groups at T4 by Student’s *t*-test with unequal variance (*p* < 0.05; *n* = 12–20/group) and between different time points (T1 to T4) by one-way ANOVA test followed by post hoc Holm multiple comparisons (https://astatsa.com/OneWay_Anova_with_TukeyHSD/; *p* < 0.05, *n* = 3–20/group). A Spearman’s Rho correlation analysis was conducted between antibody titers and *M. marinum* infection RNA levels (https://www.socscistatistics.com/tests/spearman/; *p* < 0.005).

### 3.12. Characterization of mRNA Levels of Selected Zebrafish Immune Response and Nutrient Metabolism Genes by RT-qPCR

Total gut RNA extracted as descried above (Section 3.10) was used for analysis. Selected zebrafish genes included immune response *chemokine receptor 6a* (*ccr6a*; NM_001099991.1), *toll-like receptor 2* (*tlr2*; NM_212812.1), *interleukin 1-beta* (*IL-1ß*; NM_212844.2), *akirin 2* (*akr2*; NM_213294.2), *complement component 3* (*C3*; NM_131243.1), *interleukin-6* (*IL-6*; JN698962), *tumor necrosis factor-alpha* (*tnf-α*; BC165066), *nuclear factor-kB* (*NF-kB*; AY163838) and nutrient metabolism *hexokinase 1* (*hk-1*; BC067330.1) [16,24]. Sequences were obtained from NCBI nucletotide database (https://www.ncbi.nlm.nih.gov/nucleotide/, accessed on 30 November 2020) and the UCSC Genome Browser on Zebrafish May 2017 (GRCz11/danRer11) Assembly (http://genome.ucsc.edu/cgi-bin/hgTracks?db=danRer11&lastVirtModeType=default&lastVirtModeExtraState=&virtModeType=default&virtMode=0&nonVirtPosition=&position=chr19%3A27019529%2D27023771&hgsid=1072595985_aaRkNS7FkPbTrWiA6ZHMUkLZ1fRT, accessed on 30 November 2020). To characterize the expression of selected genes, an RT-qPCR was performed for the analysis of *D. rerio* mRNA levels. The RT-qPCR was performed, and data were normalized as described above for mycobacterial RNA levels, using specific primers and conditions following manufacturer’s recommendations (Table 4). The RNA normalized Ct values were compared between groups at T4 by one-way ANOVA test followed by post hoc Holm multiple comparisons (https://astatsa.com/OneWay_Anova_with_TukeyHSD/; *p* < 0.05, *n* = 3–20/group) and between T1 and T3/T4 by Student’s *t*-test with unequal variance (*p* < 0.05; *n* = 12–20/group).

### 3.13. Characterization of Serum Total Antioxidant Capacity

Serum total antioxidant capacity (Ta) was characterized by using the potassium permanganate method [55]. Sera were diluted (1:10, 1:20, 1:40, 1:80 and 1:160) with distilled water, and 20 μL per well was added to a 96-well ELISA plate with blank no serum control. Then, 100 μL of 5 mmol/L solution of KMnO_4_ (79 mg KMnO_4_ dissolved in 100 mL distilled water) was added to each well and mixed with serum samples. Plates were incubated for 30 min at 37 °C in a water bath, after which the o.d. was measured in a spectrophotometer at 570 nm. The Ta was calculated:

Ta = 100/(OD1 + 2 × (OD2 + OD3 + OD4) + OD5) (1)

where OD1 to OD5 are the o.d. at 1:10 to 1:160 serum dilutions. Ta values were compared between treated and control groups at T4 (end of the trial) by Student’s *t*-test with unequal variance (*p* < 0.05; *n* = 14–20/group).

### 3.14. Characterization of The Zebrafish Gut Microbiome

#### 3.14.1. DNA Extraction, Amplicon Preparation, and Sequencing

A total of 39 zebrafish gut samples were selected to obtain a representative sample of each group (Group A: commercial feed with probiotic *A. veronii*, *n* = 10; Group B: commercial feed with probiotic *P.* entomophila, *n* = 11; Group C: commercial feed with PBS, *n* = 8; Group D: commercial feed with α-Gal immersion, *n* = 10) at two different time points to test whether challenge with *M. marinum* has an impact on zebrafish gut microbiota (pre-challenge with *M. marinum* at weeks 3–5, *n* = 21; post-challenge with *M. marinum* at week 10, *n* = 18) (Table 5).

Genomic DNA was extracted from individual zebrafish gut samples by using the AllPrep DNA/RNA/Protein kit. DNA sequencing was performed at the Genomic Unit Campus Moncloa (University Complutense of Madrid, Madrid, Spain). An aliquot of each DNA sample was used to prepare the libraries to amplify the V3 and V4 hypervariable regions of the *16S rRNA* gene by using the pair of primers 341F: 5′ TCGTCGGCAGCGTCAGATGTGTATAAGAGACAGCCTACGGGNGGCWGCAG and 805R:5′GTCTCGTGGGCTCGGAGATGTGTATAAGAGACAGGACTACHVGGGTATCTAATCC and PCR amplification of the amplicon target following the manufacturer’s recommendations. The expected size of the PCR products (approximately 550 bp) was verified on a Bioanalyzer DNA 1000 chip (Agilent Technologies, Santa Clara, CA, USA) and further purified by using AMPure XP beads (Beckman Coulter, Life Sciences, Pasadena, CA, USA) for further processing. Then, Illumina sequencing adapters and index barcodes, using Nextera XT DNA library preparation kit (Illumina, Inc., San Diego, CA, USA), were added to the amplicon target before libraries were pooled together for further sequencing. All cluster generation and paired-end sequencing were performed on the Illumina Next-Generation Sequencing MiSeq system, using Illumina MiSeq v2 2 × 300 cycle chemistry, following the manufacturer’s protocols.

#### 3.14.2. Downstream Data Analysis for 16S rRNA Sequencing Processing and ASVs Workflow

A total of 12,799,596 MiSeq reads passing filter were pair-end demultiplexed and fastq file generated, using the Illumina MiSeq Reporter software. The raw *16S rRNA* sequences were uploaded to the Sequence Read Archive (SRA) repository (https://www.ncbi.nlm.nih.gov/sra; BioProject ID PRJNA728442, accession numbers SAMN19079379–SAMN19079417). Sequence analysis was performed by using DADA2 inference algorithm on primer-free reads to correct sequencing errors and create the ASVs for the zebrafish gut microbial communities (v.1.12) in R (v.4.0.1) [92]. The reads were quality filtered by using the filterAndTrim (https://rdrr.io/bioc/dada2/man/filterAndTrim.html) function that truncated the forward and reverse reads at 280 bp and 255 bp for the zebrafish gut microbiota dataset. Then, reads with more than 2 errors in the forward and 2 errors in the reverse reads were removed. Reads were merged after inference of sequence variation with learnErrors (https://rdrr.io/bioc/dada2/man/learnErrors.html) and denoised by using dada (https://rdrr.io/bioc/dada2/man/dada.html) functions. Chimeric sequences were eliminated with removeBimeraDenovo (https://rdrr.io/bioc/dada2/man/removeBimeraDenovo.html), and taxonomy was assigned to ASVs by using the classify–learn naïve Bayes taxonomic classifier assignTaxonomy (https://rdrr.io/bioc/dada2/man/assignTaxonomy.html) based on the SILVA database (https://www.arb-silva.de; v.138) database [93]. Taxa count abundances were extracted from original outputs for each taxonomic level (Supplementary Materials File S1: Data S1). Microbial community profiles were constructed at kingdom, phylum, class, order, family, genus for further analysis. All algorithms and databases were accessed in 31 March 2021.

#### 3.14.3. Statistical Analysis of Gut Zebrafish Microbial Communities

For the zebrafish gut microbiota dataset, the ASVs count table was generated. A total of 8922 ASVs were assigned to the 39 samples (Table 5) and at 6 taxonomic ranks (kingdom, phylum, class, order, family and genus). All the subsequent biological analyses were performed by using the phyloseq (https://bioconductor.org/packages/release/bioc/html/phyloseq.html; v.3.10) [94] package and ggplot2 (https://ggplot2.tidyverse.org) was used for visualizations in R (v.4.0.1). All ASVs with low counts and those with prevalence lower than 0.01% were filtered to remove spurious ASVs in the bacterial dataset. Then the microbial community composition was represented in terms of relative abundance at phylum, family and genus levels, keeping the most abundant five featured taxa at each level by using the tax-glom function (https://rdrr.io/bioc/phyloseq/man/tax_glom.html) in the phyloseq package (v.3.10). For estimating microbial community dissimilarities, Bray–Curtis distances were calculated by phyloseq (v.3.10) and vegan (https://cran.r-project.org/web/packages/vegan/index.html; v.2.5.7) [94] package implemented in R (v.4.0.3). Data were normalized by rarefaction, with no replacement, using the phyloseq function rarefy_even_depht (https://rdrr.io/bioc/phyloseq/man/rarefy_even_depth.html) and clr transform, using the microbiome package (https://microbiome.github.io/tutorials/; v.1.12.0) prior to diversity measures. Further, principal component analysis (PCA) plots were constructed to visualize the categorical partition of the samples explained by Bray–Curtis dissimilarity. Adonis from vegan package (https://rdrr.io/rforge/vegan/man/adonis.html) in R was used for Permutational multivariate analysis of variance (PERMANOVA) test to evaluate differences among groups (number of permutations set at 999). The taxa differential abundance analyses were performed by using the function aldex in ALDEx2 (https://bioconductor.org/packages/release/bioc/html/ALDEx2.html; v.1.22.0) after technical filtering of ASVs with less than 5 reads in total and appearing in less than two samples [95]. Differential abundance in the zebrafish gut microbiome was assessed for all the experimental groups treated with *A. veronii* probiotic feed, *P. entomophila* probiotic feed and α-Gal versus the control group treated with PBA at each time point (pre-challenge and post-challenge). Correlations between zebrafish gut microbiota and anti-α-Gal and P22 IgM antibody titers were calculated with ALDEx2 (v.1.22.0), using the aldex.corr function analysis (https://rdrr.io/bioc/ALDEx2/man/aldex.corr.html) as implemented in R (v.4.0.3), and visualized by using the package ggplot2. All packages and algorithms were accessed on 30 March 2021.

## 4. Conclusions

Treatment with probiotics prepared with bacteria from the gut microbiota with high α-Gal content protected against mycobacteriosis in the zebrafish model of tuberculosis. This study provided the first evidence on the effect of probiotics with high α-Gal content on eliciting protection against mycobacteriosis. The main limitations of the study are the limited number of samples included in some analyses and the need to corroborate in future studies the suggested protective mechanisms elicited by probiotics with high α-Gal content. The results provided preliminary evidence that the protective mechanisms induced in response to probiotics with high α-Gal content include B-cell maturation, antibody-mediated opsonization of mycobacteria, FcR-mediated phagocytosis, macrophage response, interference with the α-Gal antagonistic effect of the TLR2/NF-kB-mediated immune response, and upregulation of pro-inflammatory cytokines and innate immunity. Additionally, a beneficial effect on nutrient metabolism was observed through upregulation of HK-1 likely in response to IL-mediated activation of MAPK. The activation of these humoral and cellular immune mechanisms reduces mycobacteria infection. Treatment with probiotic *A. veronii* and *P. entomophila* activated different mechanisms, but all associated with the response to α-Gal. While probiotic *A. veronii* with highest α-Gal content promoted B-cell maturation, only probiotic *P. entomophila* produced beneficial effects on nutrient metabolism through HK-1 and reduced oxidative stress. Remarkably, both probiotic bacteria induced innate immune response trough TNF-α upregulation. These results support the potential of probiotics with high α-Gal content for the control of fish mycobacteriosis and provided additional evidence of the role of immune response to α-Gal for the control of infectious diseases caused by pathogens with this modification on their surface. The suggested mechanisms activated in response to probiotics with high α-Gal content need to be corroborated by using other experimental approaches to characterize innate immunity or humoral and cellular immune response. Differences in the activated protective mechanisms and gut microbiota composition between probiotics and α-Gal suggested the possibility of exploring the development of combined probiotic treatments alone and in combination with α-Gal for the control of mycobacteriosis and other infectious diseases.

## Figures and Tables

**Table 1:** Phenotypic characteristics and classification of cultured bacteria isolated from zebrafish gut microbiota.

Organism	Colony Description	Classification
**Aerobic**	circular, pink, raised, punctiform bacterial colonies	Type I
	circular, creamy white, raised, bacterial colonies (≤5 mm)	Type II
	irregular, dry white, flat colonies (≤5 mm)	Type III
**Anaerobic**	circular, creamy white, raised colonies (≤5 mm)	Type Ib
	circular, white, raised, punctiform colonies	Type IIb

**Table 2:** BLAST results of *16S rRNA* gene sequences from aerobic bacterial type I colony isolates.

ID	BLAST Match to 16S rRNA	Max Score, Total Score, Query Cover, Identity, E-Value	Genebank Accession Number	References in Zebrafish
PSZ1	*Aeromonas veronii* strain JCM 7375	813, 813, 91%, 99.3%, 0.0	NR_112838.1 NR_118947.1 NR_044845.1	[37,38,39]
PSZ4	*Microbacterium mitrae* strain M4-8	773, 773, 91%, 99.1%, 0.0	NR_104520.1	[37]
PSZ9	*Dyadobacter alkalitolerans* strain 12116	778, 778, 92%, 98.4%, 0.0	NR_044476.1	[39]
LRZ3	*Shewanella xiamenensis* strain S4	826, 826, 91%, 99.8%, 0.0	NR_116732.1	[37,39]
LRZ9	*Pseudomonas entomophila* L48	826, 826, 92%, 99.8%, 0.0	NR_102854.1	[37,38,39]

The maximum identities of all V3/V4 16S rRNA gene sequences were searched by using the GenBank DNA sequence database and the BLASTN.

**Table 3:** List of significant differentially represented bacterial taxa in zebrafish microbiota.

α-Gal vs. Control at Pre-Challenge						
**Taxon**	**Diff.btw**	**Diff.win**	**Effect**	**Overlap**	**We.ep**	**We.eBH**
*Roseomonas*	13.542346	2.9935668	4.663723	0.000233886	3.212340e-04	0.02402259
*Tabrizicola*	5.322295	0.9551066	5.490361	0.000233886	1.855949e-05	0.00295325
***P. entomophila* Probiotic Treatment vs. Control at Post-Challenge**						
**Taxon**	**Diff.btw**	**Diff.win**	**Effect**	**Overlap**	**We.ep**	**We.eBH**
*Barnesiella*	−2.124928	0.4827484	−3.883899	0.000140345	0.0003810454	0.02501155
*Defluviicoccus*	−2.790908	0.9249851	−3.087608	0.000140345	0.0003750165	0.02514513
*Arenimonas*	−1.797804	0.6606933	−2.640389	0.000140345	0.0006246535	0.03367145
*Bradyrhizobium*	−1.208369	0.5903415	−2.048345	0.000140345	0.0017745113	0.04961546
*Gemmobacter*	12.002481	3.4395137	3.290789	0.000140345	0.0019083535	0.04779711
*Rubrivivax*	10.355734	2.8742810	3.437225	0.000140345	0.0017269486	0.03403588
*Dinghuibacter*	8.695993	2.4873367	3.648279	0.000140345	0.0027296612	0.04676323
*Candidatus Berkiella*	9.832489	2.5879052	3.665712	0.000140345	0.0016078763	0.03464549
*Tabrizicola*	10.649151	2.5152927	4.325241	0.000140345	0.0020747599	0.03980276

The results were obtained by using the AlDEx2 algorithms. Abbreviations: diff.btw, median difference between groups on a log base 2 scale; diff.win, largest median variation within groups; effect, effect size of the difference, median of diff.btw/diff.win; overlap, confusion in assigning and observation; we.ep, expected value of the Welch Test value; we.eBH, expected value of the Benjamini–Hochberg corrected *p*-value.

**Table 4:** Oligonucleotide primer sequences an annealing condition.

Genes	Oligonucleotide Forward (F) and Reverse (R) Primers	Annealing Conditions	References
*ccr6a*	F: 5′-AGCTTCTGCGTGGCATCTAT-3′ R: 5′-CAGACGGCTGCACAAACTAA-3′	56 °C, 30 s	[24]
*tlr2*	F: 5′-TGAATGGGTCGAGGAGATTC-3′ R: 5′-CACAAAGTGCTCCGACAGAA-3′	56 °C, 30 s	[24]
*akr2*	F: 5′-ACTATGGACTTCGATCCGCT-3′ R: 5′-GCTCTGTGGTGAGTGCTGAA-3′	56 °C, 30 s	[24]
*IL-1ß*	F: 5′-GCATGTCCACATATGCGTCG-3′ R: 5′-GCTGGTCGTATCCGTTTGGA-3′	58 °C, 30 s	[24]
*C3*	F: 5′-ACGCTCTCTGGATTGAAACA-3′ R: 5′-TGCCTTCTTGCATGGCAATC-3′	56 °C, 30 s	[24]
*IL-6*	F: 5′-TCAACTTCTCCAGCGTGATG-3′ R: 5′-TCTTTCCCTCTTTTCCTCCTG-3′	56 °C, 30 s	[16]
*tnf-α*	F: 5′-AAGGAGAGTTGCCTTTACCG-3′ R: 5′-ATTGCCCTGGGTCTTATGC-3′	54 °C, 30 s	[16]
*NF-kB*	F: 5′-AAGAGGACCAAAATAAGCACAG-3′ R: 5′-AAGTCCAAGGTACATCGCCATGA-3′	58 °C, 30 s	[16]
*hk-1*	F: 5′-ACTTTGGGTGCAATCCTGAC-3′ R: 5′-AGACGACGCACTGTTTTGTG-3′f	56 °C, 30 s	[16]

**Table 5:** Description of samples per treatment group and time point.

Time Point	Treatment			
	*A. veronii*	*P. entomophila*	α-Gal	PBS Control
**Pre-Challenge (weeks 3–5)**	*n* = 6	*n* = 6	*n* = 6	*n* = 3
**Post-Challenge (week 10)**	*n* = 4	*n* = 5	*n* = 4	*n* = 5

## Data Availability

The data presented in this study are available in the article and Sequence Read Archive (SRA) repository (https://www.ncbi.nlm.nih.gov/sra; BioProject ID PRJNA728442, accession numbers SAMN19079379-SAMN19079417).

## Supplementary Materials

The following are available online at https://www.mdpi.com/article/10.3390/ph14070635/s1. Figure S1: Expression of zebrafish immune-response genes in response to α-Gal and probiotic bacteria. File S1: Data S1. Raw counts of taxonomic assignments per sample generated with DADA2 and grouped by time point and treatment group. File S1: Figure S2. Relative abundance of the top 5 phyla. File S1: Figure S3. Relative abundance of the top 5 families. File S1: Figure S4. Relative abundance of the top 5 genera.
